# Copy Number Variation of Human Satellite III (1q12) With Aging

**DOI:** 10.3389/fgene.2019.00704

**Published:** 2019-08-07

**Authors:** Elizaveta S. Ershova, Elena M. Malinovskaya, Marina S. Konkova, Roman V. Veiko, Pavel E. Umriukhin, Andrey V. Martynov, Sergey I. Kutsev, Natalia N. Veiko, Svetlana V. Kostyuk

**Affiliations:** ^1^Research Centre for Medical Genetics (RCMG), Moscow, Russia; ^2^I.M. Sechenov First Moscow State Medical University (Sechenov University), Moscow, Russia; ^3^P.K. Anokhin Institute of Normal Physiology, Moscow, Russia

**Keywords:** copy number variance, satellite DNA, aging, replicative senescence, genotoxic stress response

## Abstract

**Introduction:** Human satellite DNA is organized in long arrays in peri/centromeric heterochromatin. There is little information about satellite copy number variants (CNVs) in aging and replicative cell senescence (RS).

**Materials and Methods:** Biotinylated pUC1.77 probe was used for the satellite III (f-SatIII) quantitation in leukocyte DNA by the non-radioactive quantitative hybridization for 557 subjects between 2 and 91 years old. The effect of RS and genotoxic stress (GS, 4 or 6 µM of K_2_CrO_4_) on the f-SatIII CNV was studied on the cultured human skin fibroblast (HSF) lines of five subjects.

**Results:** f-SatIII in leukocyte and HSFs varies between 5.7 and 40 pg/ng of DNA. During RS, the f-SatIII content in HSFs increased. During GS, HSFs may increase or decrease f-SatIII content. Cells with low f-SatIII content have the greatest proliferative potential.

F-SatIII CNVs in different individuals belonging to the different generations depend on year of their birth. Children (born in 2005–2015 years) differed significantly from the other age groups by low content and low coefficient of variation of f-SatIII. In the individuals born in 1912–1925 and living in unfavorable social conditions (FWW, the Revolution and the Russian Civil War, SWW), there is a significant disproportion in the content of f-SatIII. The coefficient of variation reaches the maximum values than in individuals born in the period from 1926 to 1975. In the group of people born in 1990–2000 (Chernobyl disaster, the collapse of the Soviet Union, and a sharp decline in the population living standard), again, there is a significant disproportion of individuals in the content of f-SatIII. A similar disproportion was observed in the analysis of a group of individuals born in 1926–1975 who in their youth worked for a long time in high-radioactive environment.

**Conclusion:** In generations that were born and who lived in childhood in a period of severe social perturbations or in conditions of environmental pollution, we found a significant increase in leukocyte DNA f-SatIII variability. It is hypothesized that the change of the f-SatIII content in the blood cells reflects the body response to stress of different nature and intensity.

## Introduction

Copy number variants (CNVs) are known to affect a large portion of the human genome and have been implicated in many diseases (Conrad et al., 2010). Satellite DNAs are tandemly repeated sequences, organized in long arrays and located in regions of peri/centromeric heterochromatin. The number of repeat units varies by several times at individual arrays. Therefore, tandem satellite DNA represents an important source of CNVs (Warburton et al., 2008; Black and Giunta, 2018; Lower et al., 2018). Satellite DNAs have a structural and regulatory role in the organization of the chromatin in the nucleus. Pericentromeric repeats perform the critical role in preserving chromosomal integrity (Plohl et al., 2008; Bierhoff et al., 2014; Brahmachary et al., 2014; Dumbovic et al., 2017). Satellite DNA instability has been associated with senescence arrest (Hédouin et al., 2016). Higher-order unfolding of satellite heterochromatin is a consistent and early event in cell senescence. Large-scale distension or “unraveling” of peri/centromeric satellites occurs in all examined models of human and mouse senescence (Enukashvily et al., 2007; Swanson et al., 2013). Some results indicate that satellite CNVs contribute to the genetic component of human longevity (Sidler et al., 2014; Nygaard et al., 2016; Zhao et al., 2018). Although whole-genome sequencing may help to identify CNVs, most analytical methods suffer from limited capacity, especially in low-mappability regions (Monlong et al., 2018). Therefore, there is little information about CNVs tandem satellite DNAs changes in the human genome with aging. Large-scale distension of satellites, reflected by the hybridization signal area increase in the interphase nuclei, may be associated with not only chromatin decondensation but also the satellite copy number increase.

Previously, we studied the fragment of the pericentromeric satellites III (f-SatIII) using a DNA probe PUC1.77 (Cooke and Hindley, 1979) specifically hybridizing to the 1q12 pericentromeric heterochromatin of the first chromosome. In lymphocytes, endothelial cells, and mesenchymal stem cells, this fragment localizes close to the nuclear membrane. Under low doses of ionizing radiation, 1q12 sites move to the nucleus center (Ermakov et al., 2009; Ermakov et al., 2010; Ermakov et al., 2011). In these studies, we noticed that 1) the area of the hybridization signal varied significantly in the donors’ lymphocytes and 2) the area of the signal was higher in elderly donors’ lymphocytes and in later passages of cultured cells. The observed differences may be explained by different levels of chromatin compaction in cells and/or by the different numbers of copies of the hybridized site 1q12 in the individual genomes. The possible variability of the f-SatIII content in the genome may also be reflected by the fact that the 1q12 area is considered to be an unstable chromatin site (Rupa et al., 1995; Sawyer et al., 1996; Sloter et al., 2007). The damage repair effectiveness in this area is also reduced (Surrallés et al., 1997). The copy number increase of that sequence was found in cancer cells by an increase of *in situ* hybridized regions number (Wong et al., 2000; Le Baccon et al., 2001; Sy et al., 2005).

Thus, one could expect 1) significant variability of f-SatIII (1q12) in the genomes of the same age people and 2) changes in this repeat content in natural and replicable aging. The generally accepted quantitative polymerase chain reaction (qPCR) method is practically not applicable for the quantitative f-SatIII analysis, since this genome area includes many tandem repeats. In addition, the damaged old cells’ DNA is a poor Taq-polymerase matrix (Korzeneva et al., 2016; Chestkov et al., 2018; Malinovskaya et al., 2018). Therefore, for the present study, we used a non-radioactive quantitative hybridization (NQH) method, which was previously tested for the human genome ribosomal tandem repeat content analysis in damaged DNA samples of old human cells (Malinovskaya et al., 2018) and cell-free circulating DNA (Korzeneva et al., 2016). As a result, for the first time, we have shown that aging is usually associated with an increase in the f-SatIII content in human cells.

## Materials and Methods

### Population Samples

The dataset included 557 individuals (412 men and 145 women) inhabiting Moscow and the Moscow region with a median age of 43 years (2–91 years). Peripheral blood samples were collected from oldest cohort subjects (80 to 91 years old) in Moscow Nursing Home for War Veterans. Blood samples from the other subjects aged 2–79 were collected in Research Centre for Medical Genetics (Moscow). The investigation was carried out in accordance with the latest version of the Declaration of Helsinki and was approved by the Regional Ethics Committee of RCMG. All adult participants signed an informed written consent to participate after the procedures had been completely explained. The written informed consent was obtained from the parents of the participants under the age of 16. Among the subjects, there were no patients with genetic diseases, which certainly shorten the lifetime.

### DNA Samples

Blood of 5 ml was collected from a peripheral vein of the subjects using a syringe flushed with heparin (0.1 ml/5 ml of blood) under strict aseptic conditions. The DNA isolation method has been described previously (Korzeneva et al., 2016; Chestkov et al., 2018; Malinovskaya et al., 2018). DNA quantification was performed in two steps: 1) initial amount of DNA in each sample was estimated by the method of UV spectroscopy; and 2) the final DNA quantification is performed fluorimetrically using the PicoGreen dsDNA quantification reagent from Molecular Probes (Invitrogen, Carlsbad, CA, United States). The DNA concentration in the sample is calculated according to a DNA standard curve.

### Nonradioactive Quantitative Hybridization (NQH)

**Figure 1:**
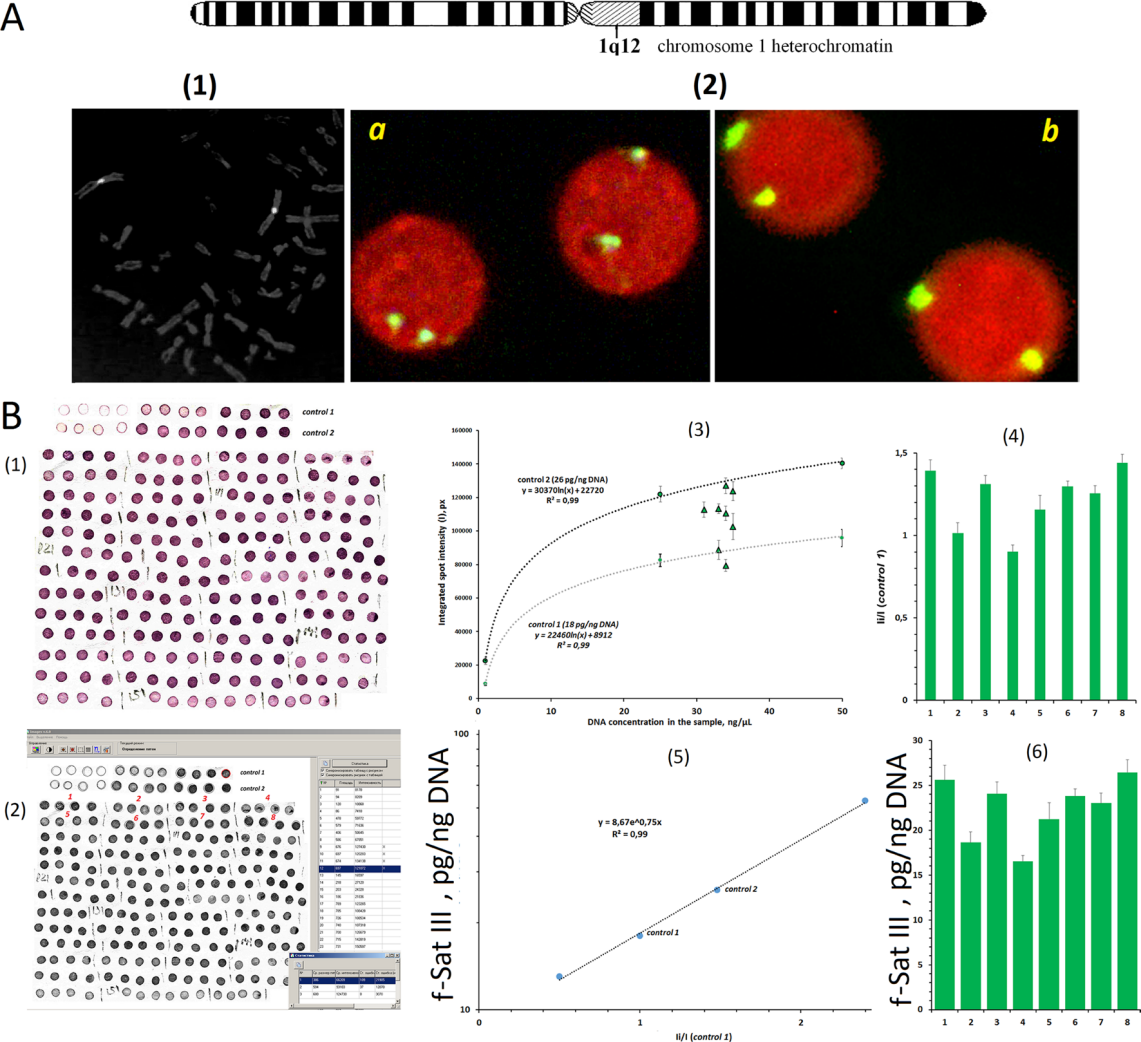
Determination of f-SatIII in human leukocyte DNA using non-radioactive quantitative hybridization (NQH). **(A)** (1) The hybridization of bio-pUC1.77 probe specifically to the lql2 region of the human chromosome 1 (f-SatIII). (2) The hybridization of the bio-pUC1.77 with the nuclei of the human lymphocytes: (a) a 17-year-old donor (6.7 pg of f-SatIII/ng DNA, detected with NQH); (b) a 77-year-old donor (31 pg of f-SatIII/ng DNA). The figures (1 and 2) show the FISH data obtained earlier in our work (Ermakov et al., 2009). **(B)** (1) Photo of the membrane fragments with visualized f-SatIII. Four spots are applied for each DNA sample. Besides, standard genomic DNA samples (control 1 and control 2) were applied onto the same filter in order to define the calibration dependence of the signal on the number of the repeats in the sample. The DNA concentration in the standard calibration sample ranged within 5–50 ng/µl. (2) The filter was scanned, and the average integral intensity *I* of the spots was determined. (3) Dependence of *I* on DNA concentration in the sample plotted for the standard samples (dashed line) and for tested DNA samples 1–8. (4) The ratio *I*_i_/*I*_control1_ was calculated. (5) Calibration dependence of f-SatIII on *I*_i_/*I*_control1_ ratio plotted for four DNA samples with known f-SatIII content. (6) The f-SatIII content in the genome was calculated. This experiment is detailed in the **Supplement**.

***The method of nonradioactive quantitative hybridization*** for tandem ribosomal repeat was specified in details previously in three publications (Korzeneva et al., 2016; Chestkov et al., 2018; Malinovskaya et al., 2018). We used this method for f-SatIII and telomere repeat analysis without modifications. The experiment is shown in **Figure 1B** and in the **Supplement**. For the calibration, we used standard human DNA samples with a known content of f-SatIII or telomere repeat (TR). This amount was previously determined by direct comparison of the content of 1.77-kb fragment (f-SatIII) or 1.1-kb fragment (TR) in a sample of genomic DNA and in a model sample that contains a known number of molecules of the analyzed fragment. Relative standard error for NQH only was 5 ± 2%. The main contribution to the overall error of the experiment is made by the step of isolating DNA from the leukocytes. The total standard error was 11 ± 7%.

***The DNA probes*.** For the detection of human telomere repeat, the following probe was used: biotin-(TTAGGG)_7_. Syntol (Russia) performed the synthesis and biotin labeling of the oligo-probe. F-SatIII probe was a 1.77-kb cloned *Eco*RI fragment of human satellite DNA (Cooke and Hindley, 1979) labeled with biotin-11-dUTP by nick translation. Dr. H. Cook (MRC, Edinburgh, UK) kindly supplied the human chromosome lql2-specific repetitive satellite DNA probe pUC1.77.

### Cell Cultures

We used five long-term lines of diploid human dermal fibroblasts. Lines HSF-41, HSF-49, HSF-57, HSF-61, and HSF-66 were obtained from skin biopsies of healthy adult persons. The digits in each line denotation indicated the order number of the final passage, after which the cells stopped their division. The cells were cultured in Eagle’s medium supplemented with 10% fetal bovine serum at 37°C at saturation humidity in an atmosphere containing 3–5% CO_2_. The cells (about 1/3 of the total amount) were subcultured approximately once every 3 days, then cultured till subconfluent condition, and subcultured again. To isolate DNA from the cells, the technique described for the blood was used. Quantification of f-SatIII and TR was performed using NQH technique.

***Genotoxic effects of (Cr(VI)).*** The method has been described previously (Veiko et al., 2005). Cells were seeded onto a 24-well plate at (30·10^3^ cells per well) and cultured for 2 days. Then 4 or 6 µM of K_2_CrO_4_ was added, cells were incubated for 24 h, the medium was replaced, and incubation was continued for up to 3 days. Cells of three to six wells were examined at certain intervals for each Cr(VI) concentration and each control variant.

***DNA fragments from the culture medium*** were isolated by phenol extraction as previously described (Veiko et al., 2005) and quantitated by fluorescence with PicoGreen. The ratio was estimated between the amount of DNA fragments isolated from the culture medium to the total amount of DNA isolated from cells and the medium.

***Caspase 3 activity*** was assayed with a Clontech kit. The activity was expressed as optical density of a *n*-nitroaniline solution per unit DNA weight after incubation of a protein lysate with the substrate at 33°C for 2 h.

***HSF-61 cell heterogeneity analysis*.** The suspension of the HSF-61 cells (passage 5) were seeded into the wells (*n* = 40) in terms of not more than 10–20 cells per well. Cells were cultured for 3 weeks, regularly changing the culture medium. An increase in the cell number was observed in 32 of the 40 wells of the plate. DNA was isolated from these wells, and its amount was measured. Further, the content of f-SatIII and TR in the DNA samples of each well was estimated by the NQH method.

### Statistical Analysis

All the findings reported here were reproduced at least two times as independent biological replicates. The significance of the observed differences was analyzed using the non-parametric Mann–Whitney *U*-test or the Kolmogorov–Smirnov statistics. Linear regression analyses were carried out to evaluate the effect of f-SatIII or telomere content in HSFs as a function of passage number. Data were analyzed with StatPlus2007 professional software (http://www.analystsoft.com/). All *p*-values were two-sided and considered statistically significant at *p* ≤ 0.01. A 1.77-kb (f-SatIII) fragment homology analysis with the human genome was performed using the “BLAST” program, and the database is presented on the website https://www.ncbi.nlm.nih.gov.

## Results

### Determination of f-SatIII Content in the Human Leukocyte DNA

The 1.77-kb cloned fragment of human satellite DNA (Cooke and Hindley, 1979) labeled with biotin (bio-pUC1.77) was used. Stringent hybridization *in situ* allows the hybridization of bio-pUC1.77 specifically to the C band lql2 [**Figure 1A**(1)]. In the lymphocyte nuclei, bio-pUC1.77 probe gave two signals [**Figure 1A**(2)]. Minor hybridization sites to the other chromosomes regions were negligible under these conditions [**Figure 1A**(1,2)]. The signal areas varied for different donors.

The NQH method determines only the total sequence content, detected by bio-pUC1.77 in the leukocyte DNA sample. Analysis of f-SatIII homology with human genome databases revealed that bio-pUC1.77 probe under severe conditions should hybridize with 1, 2, 7, 10, 16, and 22q11 chromosomes sites. However, the number of f-SatIII copies in the lql2 region is significantly more than that on other chromosomes.

The scheme of the f-SatIII CNV analysis by the NQH method is shown in **Figure 1B**. The NQH method is described in detail in the **Supplement**. The data are presented as the number of picograms of the analyzed 1.77-kb human satellite DNA fragment in 1 ng of the total DNA sample.

### The Dependence of the f-SatIII CNV in Human Leukocytes from a Person’s Age

**Figure 2:**
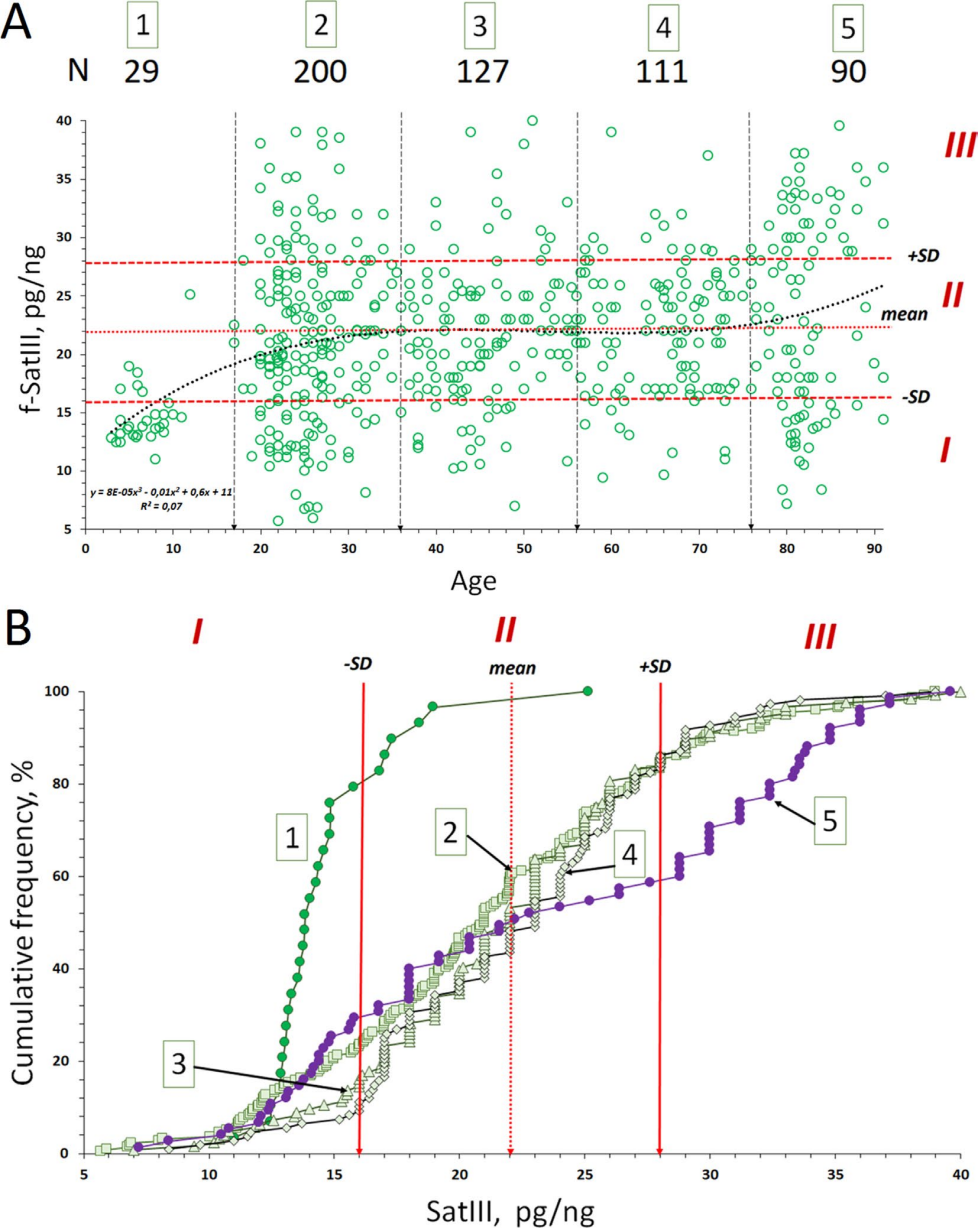
Copy number variants (CNVs) of f-SatIII in the human blood leukocyte DNA samples with age. **(A)** Association of the f-SatIII content with the age for the 557 subjects aged 2 to 91 years. The graph shows the area with low (I), moderate (II), and high (III) f-SatIII contents. All the individuals were divided into five age groups (**Table 1A**). **(B)** Cumulative distribution of the f-SatIII content in groups 1–5 of individuals of different age. Distributions for children (group 1) and for the elderly (group 5) are significantly different from those of the other groups (**Table 1B**).

The f-SatIII content in the genomes of 557 people of different age and sex varies from 5.7 to 40.0 pg/ng of DNA (mean 22.3 ± 6.9 pg/ng; median 22.0 pg/ng; **Table 1A**). **Figure 2A** shows CNV of f-SatIII in leukocytes of humans with different age. The f-SatIII content in the genome, at a first glance, weakly depends on a person’s age. The dependence is best described by a third-degree polynomial (**Figure 2A**). We found no difference in the f-SatIII content between males and females (*p* > 0.01).

**Table 1A:** Descriptive statistics for the f-SatIII content in the DNA samples of the age groups.

Group	Age range, year	*N*	Mean pg/ng	SD pg/ng	Range pg/ng	Median pg/ng	Coefficient of variation
**1**	2–12	29	14.7	2.7	11.0–25.1	13.8	0.18
**2**	17–36	200	21.2	7.2	5.7–39.0	21.0	0.34
**3**	37–56	127	21.7	6.0	7.0–40.0	21.0	0.28
**4**	57–76	111	22.2	6.0	9.4–39.0	23.0	0.25
**5**	77–91	90	23.5	8.5	7.2–39.6	23.0	0.36
**All**	2–91	557	22.3	6.9	5.7–40.0	22.0	0.32

Further, the total group was divided into five age groups (**Figure 2A** and **Table 1A**). The cumulative distribution was built for each age group (**Figure 2B**). **Table 1A** shows the descriptive statistics data for the age groups. **Table 1B** presents the results of f-SatIII content comparison between the groups with nonparametric statistics. The proportion of DNA samples with low (<16 pg/ng), high (>28 pg/ng), and average f-SatIII content (regions I, III, and II, respectively, in **Figure 2**) were determined in each group (**Figure 2B**). In the children group, DNA samples with a high content of f-SatIII (type III) were absent. A group of young donors (17–36 years old) included 25% of individuals with low (type I) and 15% with high f-SatIII contents. In the next two groups (age 37–76 years old), the number of individuals with low f-SatIII content decreased to minimum (8–10%). In this case, the number of medium-copy DNA samples (type II) increased; meanwhile, the number of high-copy samples remained at the same level. In the elderly group (77–91 years old), the number of low-copy samples increased again to 30%, while the number of samples with a high f-SatIII content in the genome increased sharply (up to 45%).

**Table 1B:** The signiﬁcance of the observed differences in the f-SatIII content in the DNA samples of the age groups was analyzed using non-parametric Mann–Whitney (*p*) and Kolmogorov–Smirnov (*D* and α) statistics.

Group X1	Group X2	X1 with X2		
		Kolmogorov–Smirnov test		*U*-test
		*D*	*α*	*p*
**1**	**2**	−0.58	4 · 10^−8^	3 · 10^−7^
	**3**	−0.66	1 · 10^−9^	2 · 10^−9^
	**4**	−0.69	2 · 10^−10^	2 · 10^−10^
	**5**	−0.57	4 · 10^−7^	4 · 10^−7^
**2**	**3**	−0.11	0.34	0.49
	**4**	−0.15	0.07	0.15
	**5**	−0.25	9 · 10^−4^	0.03
**3**	**4**	−0.11	0.41	0.41
	**5**	−0.30	1 · 10^−4^	0.14
**4**	**5**	−0.27	9 · 10^−4^	0.26

F-SatIII content in the children group is significantly lower than in other age groups (*p* << 0.01). The distribution of f-SatIII in the group of children is significantly different from other distributions. The variation coefficient values for the children group are minimal (**Figure 2B** and **Tables 1A**, B).

Elderly group (77–91 years old) by f-SatIII content does not differ significantly from other adults groups (17–76 years old) (*p* > 0.01, **Figure 2B** and **Tables 1A**, B). However, the f-SatIII content distribution in this group is significantly different from that of other groups (Kolmogorov test, **Figure 2B** and **Tables 1A**, B). The maximal variation coefficient value was found for the people of the oldest group (**Table 1A**).

These facts reflect disproportion of f-SatIII content in human genomes with age.

The dependence of the f-SatIII CNV in human leukocytes from a person’s year of birth.

**Figure 3:**
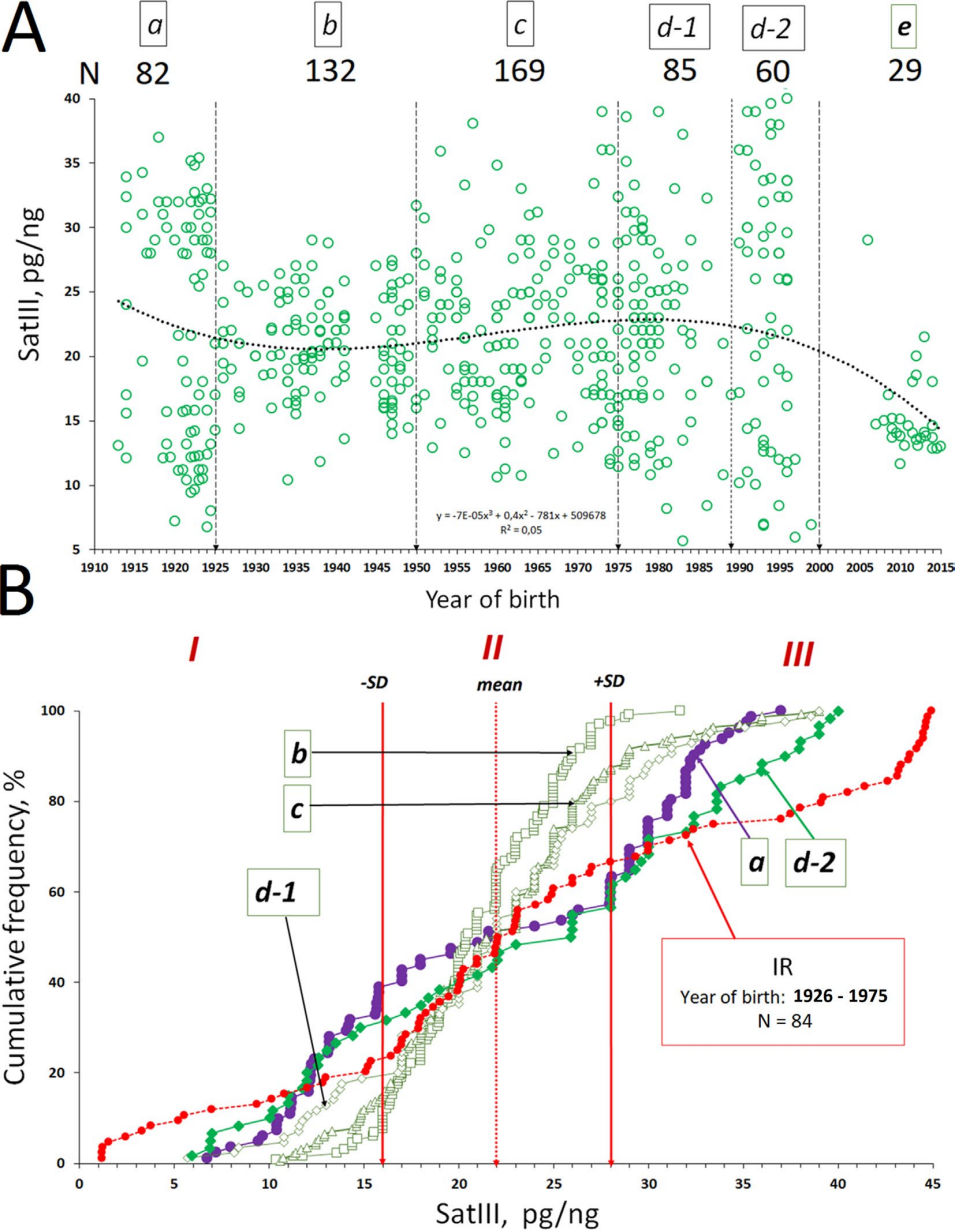
The dependence of the f-SatIII CNV in human leukocytes from a person’s year of birth. **(A)** Association of the f-SatIII content with a person’s year of birth for the 557 subjects. The total group of the adults who were born in the 20th century was divided into five groups (a–d, **Table 2A**). **(B)** Cumulative distribution of the f-SatIII content in groups a–d. Red line (group IR) shows f-SatIII content in the genomes of the adults who were born in 1926–1975. These individuals have worked for a long time with ionizing radiation sources. We published these data earlier (Korzeneva et al., 2016). Distributions for group a (1912–1925 year of birth), group d-2 (1990–2000), and IR group (1926–1975) are significantly different from those of the other groups (**Table 2B**).

The dependence of f-SatIII CNV on the time of the individual birth is shown in **Figure 3A**. The total group of the adults who were born in the 20th century was divided into five groups (**Figure 3A**): group a (1912–1925 year of birth), group b (1926–1950), group c (1951–1975), and groups d-1 and d-2 (1976–1989 and 1990–2000, respectively). Cumulative distribution (**Figure 3B**) and the variation coefficient were determined for each group (**Table 2A**).

**Table 2A:** Descriptive statistics for the f-SatIII content in the DNA samples of the groups formed by the year of birth of the individuals.

Group	Year of the birth range	*N*	Mean pg/ng	SD pg/ng	Range pg/ng	Median pg/ng	Coefficient of variation
**a**	1912–1925	82	22.0	9.0	6.7–37.0	21.6	0.41
**b**	1926–1950	132	21.0	3.9	10.4–31.7	20.7	0.19
**c**	1951–1975	169	22.0	5.8	10.6–39.0	22.0	0.27
**d-1**	1976–1989	85	22.0	7.2	5.7–39.0	22.0	0.33
**d-2**	1990–2000	60	23.6	10.3	5.9–40.0	26.0	0.44
**e**	2005–2015	29	14.7	2.7	11.0–25.1	13.8	0.18
**IR**	1926–1975	84	24.3	12.9	1.2–44.9	22.5	0.53

The f-SatIII contents in the genomes of individuals from groups a–d do not differ significantly (*p* > 0.01, **Figure 3B** and **Table 2B**). However, distributions in groups a and d-2 differ significantly from distributions in groups b, c, and d-1. Maximal variation coefficient values are in groups a and d2 (**Table 2A**). Groups a and d-2 include 35–40% of individuals with a low f-SatIII content in the genome (region I) and 45% of individuals with a high f-SatIII content in the genome (region III). Group b, c, and d-1 include 6–18% of individuals with a low f-SatIII content in the genome, and 3% to 22% of individuals with a high f-SatIII content in the genome (**Figure 3B**).

**Table 2B:** The signiﬁcance of the observed differences in the f-SatIII content in the DNA samples of the groups formed by the year of birth of individuals was analyzed using non-parametric Mann–Whitney (*p*) and Kolmogorov–Smirnov (*D* and α) statistics.

Group X1	Group X2	X1 with X2		
		Kolmogorov–Smirnov test		*U*-test
		*D*	*α*	*p*
**a**	**b **	0.41	3·10^−8^	0.48
	**c **	0.31	5·10^−5^	0.90
	**d-1**	0.23	0.01	0.91
	**d-2**	−0.16	0.28	0.36
	**IR**	−0.24	0.02	0.25
**b**	**c **	−0.17	0.03	0.18
	**d-1**	0.23	0.02	0.91
	**d-2**	−0.43	3·10^−7^	0.06
	**IR **	−0.33	2·10^−5^	0.08
**c**	**d-1**	−0.11	0.53	0.86
	**d-2**	−0.32	2·10^−4^	0.19
	**IR **	−0.26	1·10^−3^	0.26
**d-1**	**d-2**	−0.25	0.02	0.26
	**IR**	−0.21	0.03	0.37
**d-2**	**IR **	−0.19	0.16	0.80

**Figure 3B** also shows f-SatIII content in the genomes of the adults who were born in 1926–1975 (IR, red line). These individuals have worked for a long time with ionizing radiation (IR) sources. We published these data earlier (Korzeneva et al., 2016). Distribution of f-SatIII content in the genomes of these people did not differ from the distribution of the repeat content in the genomes of groups a and d-2 but significantly differed from the f-SatIII content distribution in the genomes of non-irradiated individuals born in the same period (in 1926–1975, groups b and c). We observed the increase of the individuals (in comparison with the control group) with a low f-SatIII DNA content and significant increase of the individuals with very high f-SatIII content.

Thus, the f-SatIII content variability in the genome is maximal for three groups: people born in 1912–1925 (group a), people born in 1990–2000 (group d-2), and people born in 1926–1975 but working for a long time in conditions of high radiation background.

### F-SatIII CNV During Replicative Skin Fibroblast Senescence

**Figure 4:**
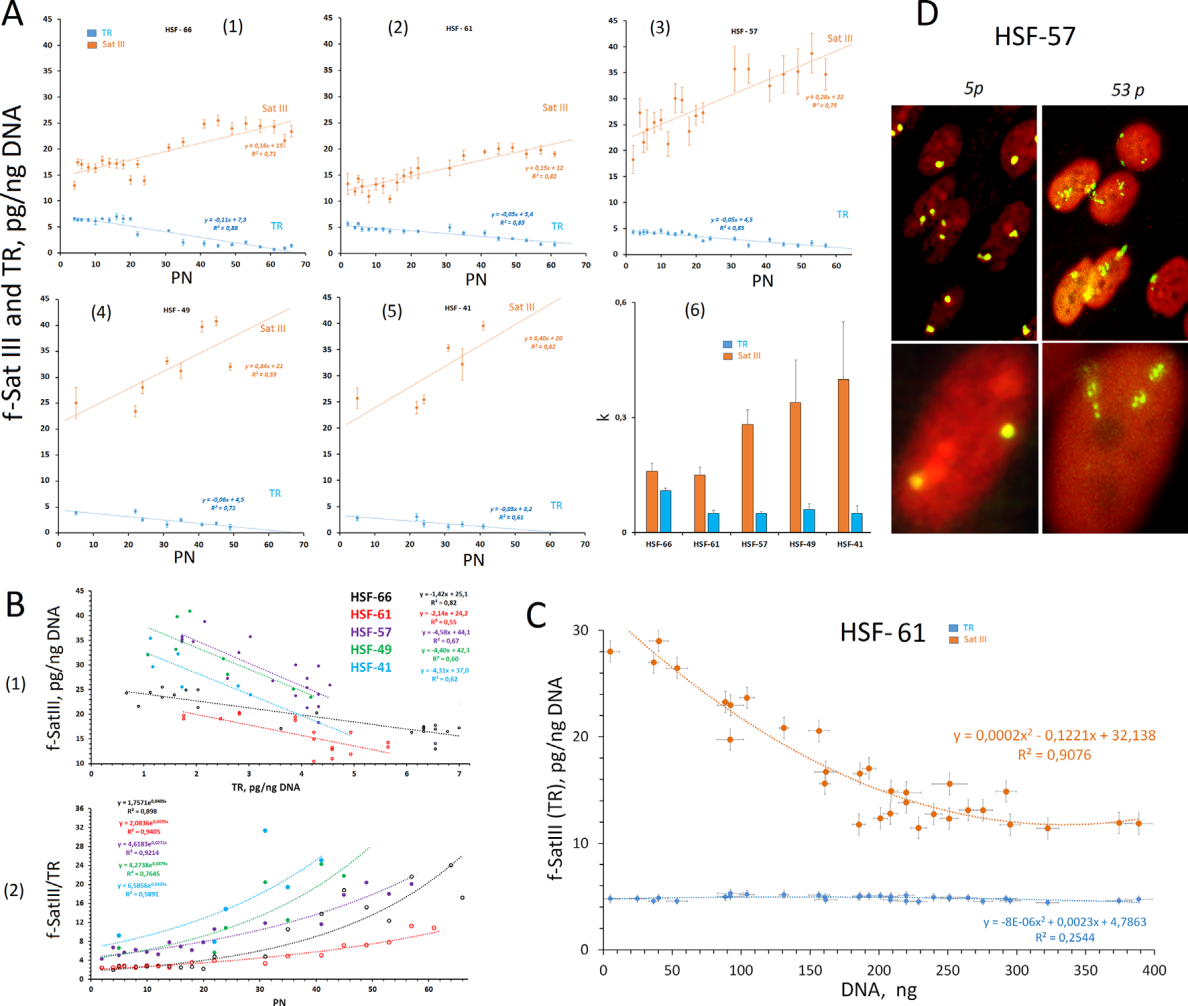
Changes in the f-SatIII content during the replicative senescence of the cultured skin fibroblasts (HSFs).** (A)** (1–5) The dependence of the f-SatIII and telomere repeat (TR) content in the genomes of HSFs on the number of passages (PN). The linear regression data are shown in the graphs. (6) The rate of change in the content of f-SatIII and TR in the genome of the HSFs during the replicative senescence. **(B)** (1) The dependence of the f-SatIII content in the genomes of HSFs on the TR content. (2) The dependence of the f-SatIII/TR ratio in the genomes of HSFs on the number of passages. **(C)** The dependence of the f-SatIII and TR content in the genomes of HSF-61 on the total cell DNA content. The suspension of the cells (fifth passage) was seeded into the wells in terms of not more than 10–20 cells per well. Cells were cultured for 3 weeks. DNA was isolated from these wells, and its amount was measured. **(D)** The hybridization of pUC1.77 probe (f-SatIII) labeled with biotin to the lql2 region of the human chromosome 1 in HSF-57 (early and late passages). The data were received and kindly provided by M.S. Konkova.

Five human skin fibroblast lines derived from primary cells of 35- (*N* = 2), 52- (*N* = 2), and 21-year-old (*N* = 1) donors were used for CNV analysis of f-SatIII in the genome during replication senescence. The cells were cultivated until they reached the limit of Hayflick and stopped dividing. The numbers that indicate the last passage number indicates fibroblast strains: HSF-66, HSF-61, HSF-57, HSF-49, and HSF-41. The NQH method was used to determine the content of two tandem repeats (f-SatIII and TR) in DNA. **Figure 4A** (1–5) shows the dependence of two repeat contents from the number of the passages. For all cell lines, we observed the f-SatIII increase in the genomes of older cells together with the expected decrease in TR.

**Figure 4A** **(6)** shows the changes in the content of repeats during the replication senescence in the form of the slope tangent of the corresponding trend lines. Cultures with the maximum number of passages (HSF-66 and HSF-61) initially contained the minimum number of f-SatIII repeat (10–15 pg/ng of DNA), and the rate of the repeat content increase during senescence was two to three times lower than that of cultures that initially contained a large amount of f-SatIII (20–25 pg/ng of DNA).

**Figure 4B** **(1)** shows the correlation between f-SatIII and TR in the fibroblast DNA. The slope of the line angle for the cultures with the maximum number of passages (HSF-66 and HSF-61) is two to three times less than for HSF-57, HSF-49, and HSF-41.

**Figure 4B** **(2)** shows the dependence of the f-SatIII/TR ratio on the number of passages. The exponent approximates the dependence. At the first 20 passages, the f-SatIII/TR ratio did not change and then increased significantly. For HSF-66 and HSF-61 cultures, this index was initially 1.5–2, and for the remaining, 4–8.

**Figure 4D** illustrates FISH data with bio-pUC1.77 probe for 5 and 53 passages of HSF-57 strain. In young cells, the signals are located compactly. For old cells, the area of signals increased significantly. An average signal area increase during aging was 3.8 ± 0.5 times, while the f-SatIII DNA content increased less than two times. Thus, the increase in the hybridization signal area in the replication senescence of fibroblasts is associated with an increase of SatIII copy number in the fibroblast genomes and with the area heterochromatin decondensation in old cells.

### Variation of f-SatIII Content in the One-Population Cells

The observed dependence of the f-SatIII content in HSFs on the number of passages may have two explanations. The repeat content may increase simultaneously in all the cells of the population or the population may increase the number of cells with a high f-SatIII content in DNA and decrease the content of cells with low amounts of repeat. To assess the possible f-SatIII DNA content heterogeneity of the HSF strain cells, we conducted a cloning experiment. The HSF-61 cells (fifth passage) suspension was sieved into the plate wells (*n* = 40) (not more than 10–20 cells per each well). The cells were cultured for 3 weeks while regularly changing the culture medium. The growth of the cell number was observed in 32 of the 40 wells. The measured DNA amount from these wells is proportional to the number of cells in each sample. The f-SatIII content was measured in each well using the NQH method. **Figure 4C** shows the dependence of f-SatIII content on the amount of cellular DNA. We found that the lower the proliferative activity of the cells in the well (the smaller the amount of DNA), the higher the f-SatIII content.

Thus, we have shown that 1) the HSF population is heterogeneous by f-SatIII content and 2) only the cells that contain low f-SatIII amount actively divide.

### CNV of f-SatIII in Fibroblasts Under Genotoxic Stress

**Figure 5:**
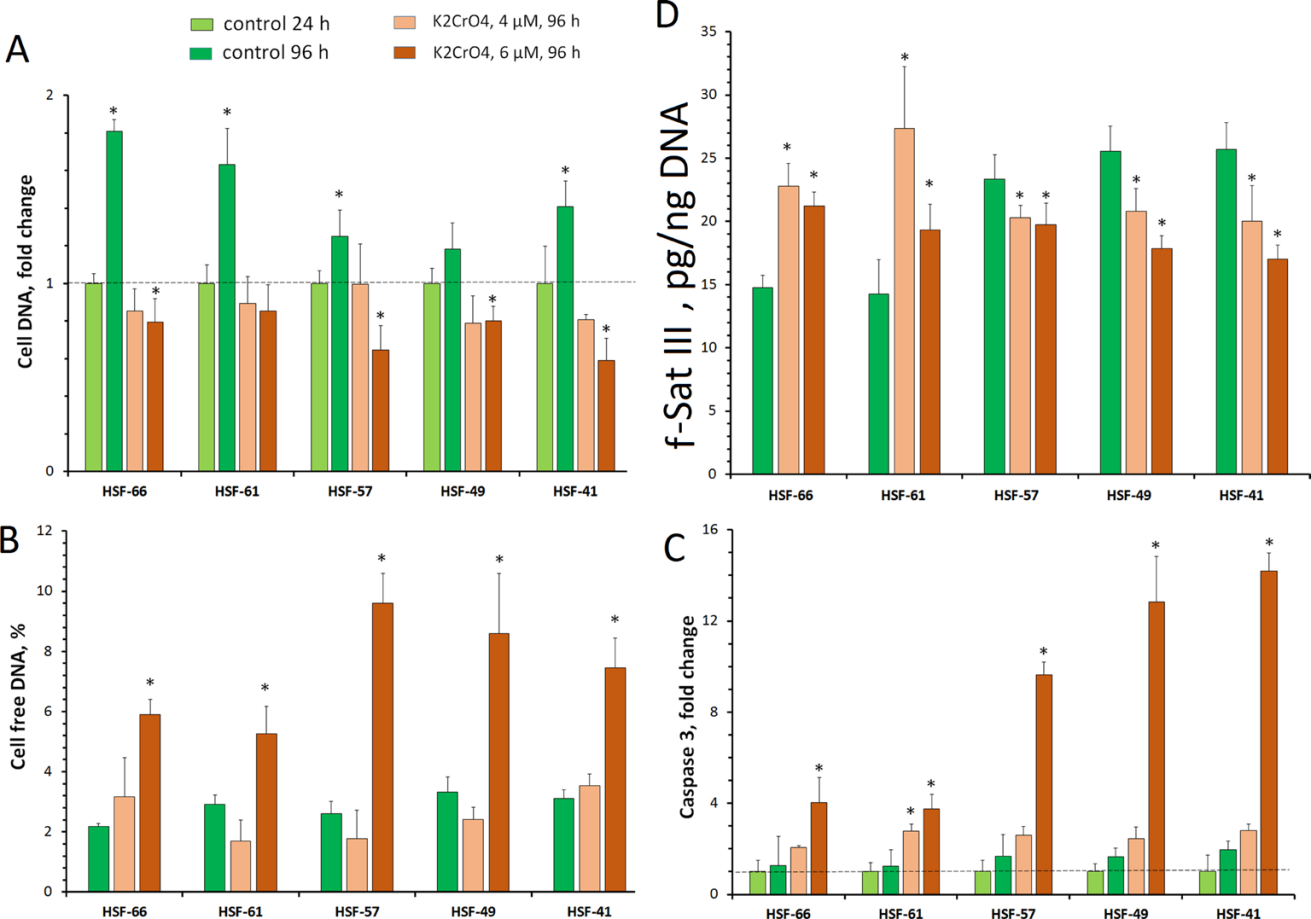
HSF response to genotoxic effect of K_2_CrO_4_. Cells were seeded onto a 24-well plate and cultured for 48 h. Then 4 or 6 µM of K_2_CrO_4_ was added, cells were incubated for 24 h (point 24 h), the medium was replaced, and incubation was continued for up to 72 h (point 96 h). Cells of three to six wells were examined for each K_2_CrO_4_ concentration and each control variant. **(A)** Change for the cell DNA on the plate. **(B)** Change for the cell-free DNA in the medium. **(C)** Change in caspase 3 activity in the protein lysate of the cells. **(D)** Change in the f-SatIII content in the DNA. *p < 0.05.

Human cell senescence is associated with genotoxic stress, leading to proliferation arrest. To assess how the f-SatIII content associates with stress intensity, we used a previously tested model with human skin fibroblasts cultured in the presence of Cr(VI). Cr(VI) induces oxidative stress with severity depending on the Cr(VI) concentration (Carlisle et al., 2000; Veiko et al., 2005; Asatiani et al., 2011; Xie et al., 2015; Monteiro et al., 2019). The fifth-passage HSF-66, HSF-61, HSF-57, HSF-49, and HSF-41 cells were cultured in the presence of 4 or 6 µM of K_2_CrO_4_ for 24 h. After the culture medium was changed to a fresh one, the cells were cultivated for the next 72 h. To assess their proliferative activity, the amount of DNA in the cells was measured (**Figure 5A**). The level of cell death was estimated by the DNA amount in the culture medium (**Figure 5B**) and the caspase 9 activity in the cells (**Figure 5C**).

Cr(VI) of 4 µM applied for 24 h caused cell damage and blocked proliferation. However, after subsequent cultivation under normal conditions, pronounced cell death did not occur. The DNA amount does not change compared with the control level (24 h). Low extracellular DNA and caspase 9 activity also indicate low level of cell death.

Cr(VI) of 6 µM induced more significant cell damage. The total number of cells decreased, and the amount of extracellular DNA and the caspase 9 activity increased.

The content of f-SatIII in DNA is shown in **Figure 5D**. Moderate stress [4 µM of Cr(VI)] induces f-SatIII content increase in the DNA of early passage cells with low repeat amount (HSF-66 and HSF-61) to about the same extent as replication senescence. Strong stress [6 µM of Cr(VI)] reduces the observed effect on these cells. Strains with a higher initial f-SatIII content (HSF-57, HSF-49, and HSF-41) in response to genotoxic Cr(VI) action reduce the f-SatIII amount in genome.

Therefore, in response to genotoxic stress, cultured cells from different donors may both increase and decrease the f-SatIII content. Cells characterized by a large number of passages with lower f-SatIII content increase the repeat content after stress. Cells with high f-SatIII content and low proliferative potential in response to additional stress reduce the amount of this repeat.

## Discussion

We investigated the CNVs of satellite III subfraction (1q12) in natural human aging and replicative cell senescence. The analyzed 1.77-kb fragment (f-SatIII) is a part of highly repeating long and complex sequences, which are located mainly in the 1q12 region and, to a lesser extent, are represented on other human chromosomes (Cooke and Hindley, 1979; van Dekken et al., 1990). The 1q12 site of the human heterochromatin belongs to the unstable human genome regions. The changes in that area are often observed in cancer cells (Rupa et al., 1995; Sawyer et al., 1996; Sloter et al., 2007). Under stress and during cell culture senescence, the changes in the structural organization of these regions of the first chromosome were found, namely, the movement from the membrane to the center of the nucleus (Ermakov et al., 2009), heterochromatin decondensation, sequence demethylation, and finally transcription within that region (Enukashvily et al., 2007).

Rearrangements of pericentromeric heterochromatin were tested using FISH method, which is not able to quantify f-SatIII CNVs in the genome. The NQH method is able to determine the total f-SatIII content in the genome (**Figure 1**). In contrast to the generally accepted qPCR method, NQH requires large amounts of DNA and is much less sensitive, limiting its applicability for low-copy genome region quantitative analysis. However, for tandem moderate repeat study, NQH is suitable, especially for old cells’ DNA damage analysis (Malinovskaya et al., 2018).

### CNV of Human Satellite III (1q12) With Aging

In the children’s genome (group 1, **Figure 2A**), the f-SatIII content is significantly reduced and varies in a very narrow range than in the rest of tested sample subjects. The f-SatIII fragment content in leukocyte genomes of young and old people varies approximately in the same range—from 5 to 40 pg/ng of DNA (**Figure 2A**). This range is narrowed for 27- to 76-year-old people, where low-copy variants are less common (**Figure 2B**). In the elderly group (77–91 years old), DNA samples containing a large number of repeat copies were much more common, and the number of low-copy variants increased.

We found that the group variability of f-SatIII content in the human genome depends on the time of birth of individuals in that group (**Figure 3A** and **Table 2**). The lowest variability of f-SatIII content was found in the group of children born in the 21st century and in the group of individuals born in 1925–1950. The coefficient of variation is gradually increasing in groups of people born in 1951–2000. The maximum variability of the repeat content in DNA was found in the group of people who were born in 1912–1925 and in 1990–2000.

To determine the reasons of the different f-SatIII content variability in the different age groups, we conducted three additional studies:

1. We compared the f-SatIII content in the control group (born in 1926–1975) and in the IR group of people (born in 1926–1975) who worked with IR sources and were exposed to low doses of IR for a long period (**Figure 3B**); the data were obtained earlier in our study (Korzeneva et al., 2016). The number of individuals with high and low repeat contents in the IR group, compared with the control group, significantly increases. Thus, additional genotoxic stress induced by radiation affects the f-SatIII content in the DNA of young and middle-aged people, causing consequences that are typical for the elderly people (born in 1912–1925) and for the young people born in 1990–2000.
2. Next, we investigated the effect of induced genotoxic stress of different intensities on the f-SatIII content in the DNA of five adult individuals’ cultured skin fibroblasts. For this purpose, we studied an effect of different amounts of the genotoxic agent K_2_CrO_4_ on fibroblasts. HSF-61 and HSF-66 strains with the highest proliferative potential and the lowest f-SatIII content after 4 µM of K_2_CrO_4_ increased the f-SatIII DNA content. An increase of the K_2_CrO_4_ concentration to 6 µM decreased the f-SatIII content in these cell genomes. The HSFs with low proliferative potential under 4 and 6 µM of K_2_CrO_4_ lost f-SatIII. Thus, moderate genotoxic stress stimulates an increase in the repeat content in the cells demonstrating high proliferative activity, and a decrease in f-SatIII content in the cells demonstrate low proliferative activity.
3. Finally, we investigated the effect of replicative senescence on the f-SatIII content in the DNA of the same cultured skin fibroblasts. Replication senescence was accompanied by an increase in the f-SatIII content in the fibroblasts, and the shorter the Hayflick limit (Hayflick, 1965) for the cells, the faster the increase of the f-SatIII content in the genome (**Figure 4A**). The cells with the highest proliferative potential (HSF-61 and HSF-66) contained minimal f-SatIII amounts in the first passages. Experiments with HSF-61 cell cloning also showed that in the early passage cell population, only the cells with low repeat content could actively divide. Thus, replication senescence increases the number of cells with high f-SatIII levels in the genomes.

It seems likely that the primary response of human cells to moderate oxidative stress is an increase in the f-SatIII repeat content. With an increase of stress above the physiological level, the cell genomes lose the f-SatIII. The response of cells depends on the genetically determined ability of a particular genome to resist oxidative stress and on the level of stress itself.

The various environmental influences and/or different social stress levels may possibly explain significant fluctuations in the f-SatIII content distributions found in different generations. For example, people of group a (1912–1925) were born during the First World War, the Russian Revolution, and the Russian Civil War. Most of the people in this group actively participated in the Second World War. In other words, the people of that generation at birth and in young age were exposed to significant stress. In the generation born between 1975 and 2000, we have identified a group of individuals (d-2) born in the 90s of the last century. In addition to radioactive contamination of the environment, including the Chernobyl accident in 1986, these people were affected by the stress associated with the social perturbations that caused the collapse of the Soviet Union. Disease and mortality level increased significantly at that period. The birth rate has been falling as infant mortality has increased. The high variability of f-SatIII content in the genomes of people born in 1990–2000 is comparable with the same index in the group of people born in 1912–1925 and in the group of irradiated people born in 1926–1975. However, further research is needed for reliable conclusions. It is necessary to analyze the f-SatIII content in the same persons’ DNA samples obtained in the different periods of their lives.

**Figure 6:**
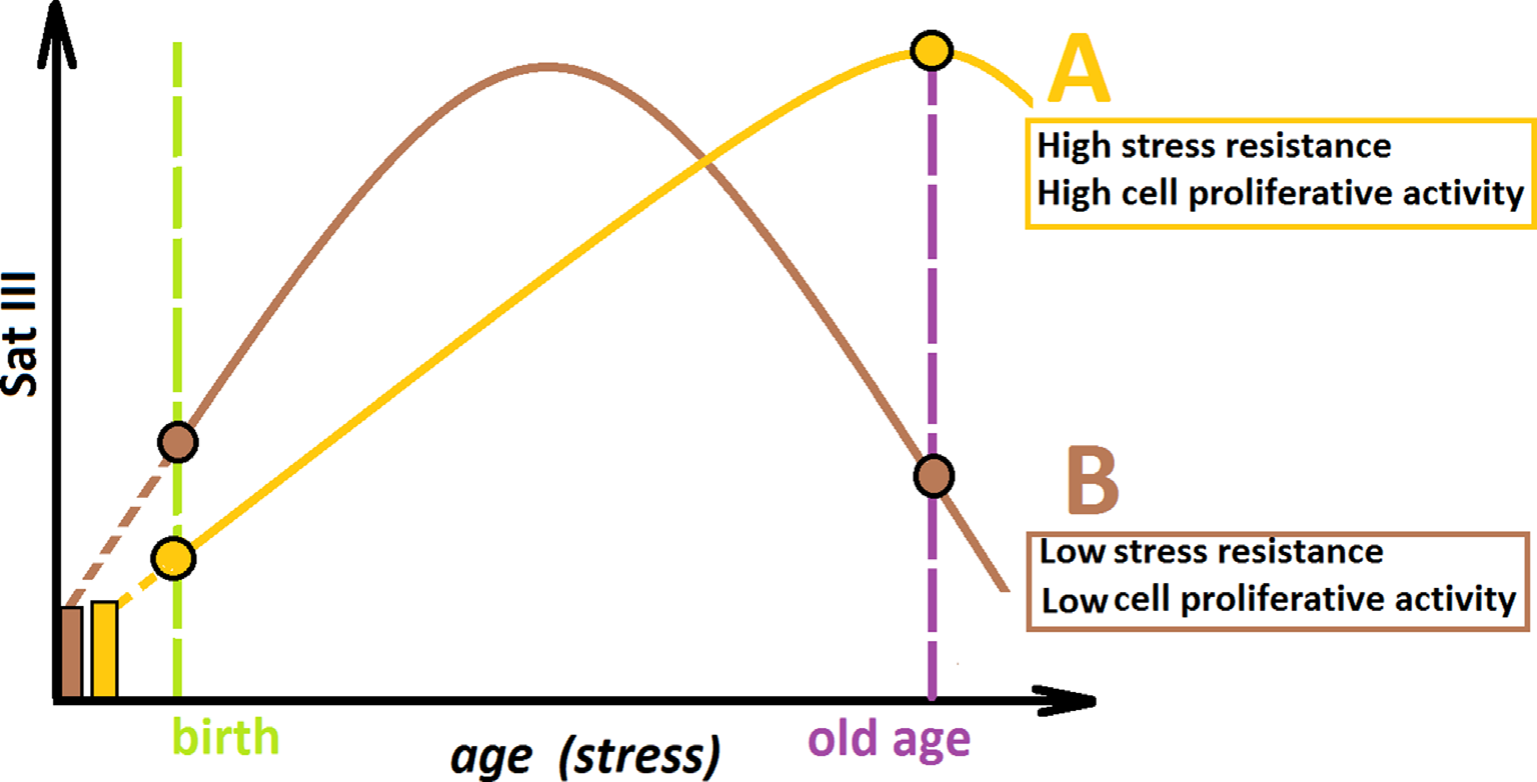
A hypothetical scheme illustrating the changes in the f-SatIII content during human aging. Individual **(A)** and individual **(B)** have the same f-SatIII repeat copy number at the time of conception. **(A)** is more resistant to genotoxic stress than **(B)**. During an aging under identical living conditions, the content of f-SatIII increases faster in **(B)**. At the young age, the f-SatIII content in B will be higher. Reaching its maximum, the f-SatIII content of **(B)** will start to decline, as aging is associated with increased stress and the cells with high f-SatIII content die. If **(B)** would live to an old age, the f-SatIII content analysis will reveal high values for **(A)** and low values for **(B)**. The proportion of the individual with high f-SatIII content in the elderly group is significantly increased. Therefore, it seems that the chances for a longer life are greater for **(A)**.

Combining the data obtained in this study, we propose the hypothetical scheme, illustrating the f-SatIII CNVs during aging (**Figure 6**). We may consider two hypothetical individuals (A and B) with the same f-SatIII repeat copy number at the time of conception. Individual A is more resistant to genotoxic stress in comparison with individual B. During aging under identical living conditions, the content of f-SatIII increases faster in the DNA of individual B in response to more pronounced stress. At the young age, the f-SatIII content in DNA of individual B will be higher. Reaching its maximum, the f-SatIII content of individual B will start to decline, as aging is associated with increased stress and the cells with high f-SatIII content die. If individual B would live to an old age, the f-SatIII content analysis will reveal high values for individual A and low values for individual B. These speculative assumptions require further experimental confirmation to answer the following remaining questions:

1. What processes reduce the f-SatIII content in DNA of individual B in old age? It may be either loss of repeat in most cells or mass death of cells with high f-SatIII content and consequent enrichment of the population with low f-SatIII content cells, which are more resistant to apoptosis and retain proliferative potential. The cloning data (**Figure 4C**) preliminarily approve that second assumption.
2. What mechanism increases the f-SatIII content in senescence cells? Perhaps this mechanism is similar to that of cancer cells. It is assumed that in cancer cells, RNA transcription from the pericentromeric satellite sequence regions is activated. Physiological induction of endogenous Sat RNA generates cDNA intermediates in the form of DNA/RNA hybrids. These hybrid molecules are stably incorporated within pericentromeric loci. Analysis revealed that human SatII copy number gain is a common feature in primary human tumors and is associated with a lower overall survival. The fact that the transcription from the Sat–genome sequences increases with normal aging of human cells as well as under genotoxic effects (Wong et al., 2001; Valgardsdottir et al., 2008; Sengupta et al., 2009; Eymery et al., 2010; Bersani et al., 2015) indicates the advantage of this mechanism in the case of senescence of non-cancer cells.
3. Is a block of proliferation and/or increased cell death a result of the f-SatIII content increase in the cell genome due to the nuclei structural organization disruption? Or is the f-SatIII increase a consequence of genome function impairment and should only be considered as another potential marker of cell proliferative activity and/or senescence (Wang and Dreesen, 2018)?

For example, it was shown that copy number polymorphism of other tandem repeats in genome (ribosomal repeat, rDNA) might be a significant source of gene expression regulatory variation in the nucleus. Polymorphisms in rDNA copy number are relevant to the maintenance of genome-wide chromatin structure. The ability of the rDNA to change in copy number provides a novel mechanism for adaptation to environmental changes by maintaining a euchromatin/heterochromatin balance (Paredes and Maggert, 2009; Paredes et al., 2011). Apparently, the polymorphisms of other genome tandem repeats may exert a similar effect on functional genome activity.

It is possible to assume that the f-SatIII content in the human genome changes not only in the process of aging or under environmental factors but also in diseases accompanied by oxidative stress. All these issues require further research.

## Data Availability

All datasets generated for this study are included in the manuscript and the **Supplementary files**.

## Ethics Statement

The investigation was carried out in accordance with the latest version of the Helsinki Declaration and approved by the Regional Ethics Committee of RCMG. All participants signed an informed written consent to participate after the procedures had been completely explained.

## Author Contributions

SK and NV designed the study; EM, EE, MK, and NV performed the experiments; RV performed the statistical analysis; PU and SIK provided the human blood samples; EM provided cell cultures; SVK and NV wrote the initial draft; PU translated the manuscript to English; all the authors participated in critical revision and approved the ﬁnal manuscript before submission.

## Funding

The Russian Science Foundation (Grant No. 18-15-00437) supported this research.

## Conflict of Interest Statement

The authors declare that the research was conducted in the absence of any commercial or financial relationships that could be construed as a potential conflict of interest.
