# Supplementary material for: Copy Number Variation of Human Satellite III (1q12) With Aging
Source: Front Genet. 2019 Aug 7;10:704. doi: 10.3389/fgene.2019.00704 (PMC6692473; doi:10.3389/fgene.2019.00704)
Supplement: Supplementary file 1 [file Table_1.docx]

**SUPPLEMENT**

***Nonradioactive quantitative hybridization (NQH)***

For the detection of the human f-SatIII repeat, 1.77-kb cloned EcoRI fragment of human satellite DNA (Cooke and Hindley 1979) labeled with biotin-11-dUTP by nick translation was used (Biotin NT Labeling Kit, Jena Bioscience GmbH).

The membrane (ExtraC) was wetted with the solution of 10хSSC and dried. One (1) µL of 1M sodium hydroxide was added to 10 µL of the DNA solution in the TE-buffer at 0°С. The solution was mixed and incubated for 10 minutes at 0°С. Then, the DNA solution was neutralized with 11 µL of 20хSSC (рН = 5.0). The denatured DNA samples were applied to a filter in the amount of 2 µL per a dot. With respect to each sample, from 3 to 6 parallel dots were applied. Besides, standard genomic DNA samples were applied onto the same filter in order to define the calibration dependence of the signal on the number of the repeats in the sample. The DNA concentration in the standard calibration sample ranged within 5 - 50 ng/µL.

The filter was heated at 80°С in vacuum for 2 hours. In order to perform the hybridization, a special equipment was used, i.e. the hybridization furnace, which can vary the temperature and has a swaying table for mixing the solution above the membrane. The membrane was fixed in the solution (Denhard 10х solution; 0.05 М phosphate buffer, рН 7.0; 0.5 М sodium chloride; 50% formamide; 100 µg/µL tRNA E.coli) for 2 hours at 42°С. Then, the denaturated DNA probe in the concentration of 20 ng/µL was added. The hybridization was conducted for 16 hours at 45°С. The filter was washed with the solution of 2хSSC, 0.1 % SDS (2х15 min, 25 °С), 0.01 × SSC, 0.1 %SDS (20 min, 65°С) and 2хSSC (10 min, 25°С). After the hybridization the membrane was fixed (30 min; 37^о^С) with a special solution (0.1% fat-free milk; 0.1% gelatin; tris-HCL buffer, рН 7.5; 0.1 М sodium chloride). Then, for 20 minutes (25°С) it was treated with the alkaline-phosphatase-conjugated streptavidin (1 µg/µl, Sigma) in a solution (0.1 М tris-HCL buffer; рН 7.5; 0.1 М sodium chloride; 0.005 М magnesium chloride). Then the filter was washed (3х10 min) with the following solution: (tris-HCL buffer рН 7.5; 0.1 М sodium chloride; 0.005 М magnesium chloride). After that, the filter was placed into the solution of substrates for alkaline phosphatase (tris-HCL buffer, рН 9.5; 0.1 М sodium chloride; 0.005 М magnesium chloride; 4.4 µg/µL NBT and 3.3 µg/µL BCIP). The reaction was performed in the darkness at 25°С, while visually controlling the appearance of colored violet spots. Upon the end of the reaction, the filter was washed with water and dried in the darkness.

The dried filter was scanned. In order to perform the quantitative analysis of the hybridization result, it was used the software application “Imager 6.0” (MGNC RAMS). The software defines the spot location, the near-by background signal, as well as the surface area and integral intensity of the spot. The signals for a single sample are averaged; the average value and standard error are defined. The f-SatIII in a particular sample is calculated using the calibration curve equation.

**Standard calibration curves.**

Six standard samples of the genomic DNA (10 - 50 ng/mL) with a known content of the f-SatIII were applied to the same filter, in order to plot a calibration curve for the dependence of the signal intensity on the f-SatIII content in a particular sample.

To obtain the calibration samples with the known content of f-SatIII, we added model f-SatIII fragment to samples of human DNA with the lowest f-SatIII content in the amount of several picograms per ng of DNA. Lambda phage DNA (10 - 50 ng/mL) was also applied to the same filter in order to control the nonspecific signal. Using this procedure, six standard samples of genomic DNA with various f-Sat III content were selected.

The dependence of the f-SatIII content on the relative hybridization signal is well reproduced in independent experiments. Therefore, for each specific experiment, two calibration samples can be used instead of six.
